# Impact of plaque and luminal morphology in balloon angioplasty of the femoropopliteal artery: an intravascular ultrasound analysis

**DOI:** 10.3389/fcvm.2023.1145030

**Published:** 2023-06-12

**Authors:** Yuchi Zou, Qiang Tong, Xuehu Wang, Chuli Jiang, Yuanbin Dai, Yu Zhao, Jun Cheng

**Affiliations:** ^1^ Department of Vascular Surgery, The First Affiliated Hospital of Chongqing Medical University, Chongqing, China; ^2^ Department of Endocrinology, The Second Affiliated Hospital of Army Medical University, Choingqing, China

**Keywords:** intravascular ultrasound (IVUS), plaque, lumen, eccentricity, angioplasty

## Abstract

**Objective:**

To assess the effect of plaque and luminal morphologies in balloon angioplasty of femoropopliteal lesions using intravascular ultrasound (IVUS).

**Methods:**

This retrospective, observational study analyzed 836 cross-sectional images using IVUS, from 35 femoropopliteal arteries of patients who underwent endovascular treatment between September 2020 and February 2022. Pre- and post-balloon angioplasty images were matched per 5 mm. Post-balloon angioplasty images were grouped into successful (*n* = 345) and unsuccessful (*n* = 491) groups. Plaque and luminal morphologies (such as severity of calcification, vascular remodeling, and plaque eccentricity) were extracted before the balloon angioplasty procedure to identify the predictors of unsuccessful balloon angioplasty. Additionally, 103 images with severe dissection were analyzed using IVUS and angiography.

**Results:**

In univariate analyses, the predictive factors for unsuccessful balloon angioplasty were vascular remodeling (*p *< .001), plaque burden (*p *< .001), lumen eccentricity (*p *< .001), and balloon/vessel ratio (*p *= .01). Predictive factors for severe dissections were the guidewire route (*p *< .001) and balloon/vessel ratio (*p *= .04). In multivariate analysis, the predictive factors for unsuccessful balloon angioplasty included lumen eccentricity (odds ratio [OR]: 3.99, 95% confidence interval [CI]: 1.28–12.68, *p *= .02) and plaque burden (OR: 1.03, 95% CI: 1.02–1.04; *p *< .001). For severe dissections, the independent risk factor was an eccentric guidewire route (OR: 2.10, 95% CI: 1.22–3.65, *p *= .01).

**Conclusion:**

High plaque burden and luminal eccentricity were risk factors for failed femoropopliteal artery balloon angioplasty. Additionally, eccentric guidewire routes predicted severe dissection.

## 1. Introduction

Femoropopliteal artery endovascular treatment requires plain balloon angioplasty. This procedure is performed after the guidewire crosses the target lesion. Residual stenosis <30% (revealed by visual inspection of the artery diameter), without flow-limiting dissection, after balloon angioplasty is considered to indicate effectiveness (1). High residual stenosis and severe dissection lead to failed plain balloon angioplasty, affecting the patient's immediate vascular patency and long-term outcomes (2–4). Studies reported that disease duration and severe calcification were risk factors for severe dissections (5, 6); however, research on the predictors of high residual stenosis preceding balloon angioplasty failure is lacking. Additionally, the impact of intravascular conditions (plaque morphology, plaque distribution, shape of the lumen, and other characteristics of the femoropopliteal artery) on the outcome of balloon angioplasty is unclear.

Angiography was used as a visual guide in lower-limb arterial endovascular treatment. Studies indicated the limitations of angiography in the measurement and assessment of vessel size, calcification, and vessel wall conditions (7, 8). Intravascular ultrasound (IVUS), commonly used in coronary angiography but rarely for femoropopliteal artery imaging, improved long-term patency of the femoropopliteal arteries because of its high vascular monitoring and measurement accuracy (9). Plaque and lumen eccentricity and vascular remodeling can be quantitatively analyzed by transversal imaging of the femoropopliteal artery using IVUS; this helps surgeons monitor vascular conditions accurately. Additionally, the automatic pulling-back device in the IVUS console solved the location mismatch problem and improved the retrospective analysis quality between the two pullbacks of the IVUS catheter (10). The relationship between plaque and stent underexpansion or guidewire routes and severe dissection have also been investigated (11, 12). However, there are no studies on IVUS-based assessment of the relationship between intravascular morphology and balloon angioplasty outcomes in lower-limb arterial endovascular treatment.

This study investigated the impact of plaque and luminal morphologies on therapeutic outcomes following plain balloon angioplasty for femoropopliteal lesions. Plaque, lumen, and arterial elastic membrane characteristics were extracted to explore their relationships with high residual stenosis and severe dissections.

## 2. Methods

### 2.1. Inclusion and exclusion criteria

Inclusion criteria were as follows: (1) endovascular therapy guided by IVUS; (2) target lesion located in the femoral or popliteal artery (P1 segment); (3) primary stenosis or occlusion disease; (4) intact angiography and IVUS data during therapy; and (5) an automated IVUS pullback device.

IVUS cross-section exclusion criteria were as follows: (1) low quality of images; (2) artefacts from the guidewire and non-uniform rotational distortion (13), causing difficulty in obtaining clear images of the vessel leading edge; (3) IVUS catheter placed at angles that cause the trailing edge of the external elastic membrane (EEM) to show 270° of the vessels in a cross-sectional image; (4) large side branches affecting EEM measurement, and (5) if the pre- and post-angioplasty cross-sectional images were not matching.

### 2.2. Study samples and design

**Figure 1:**
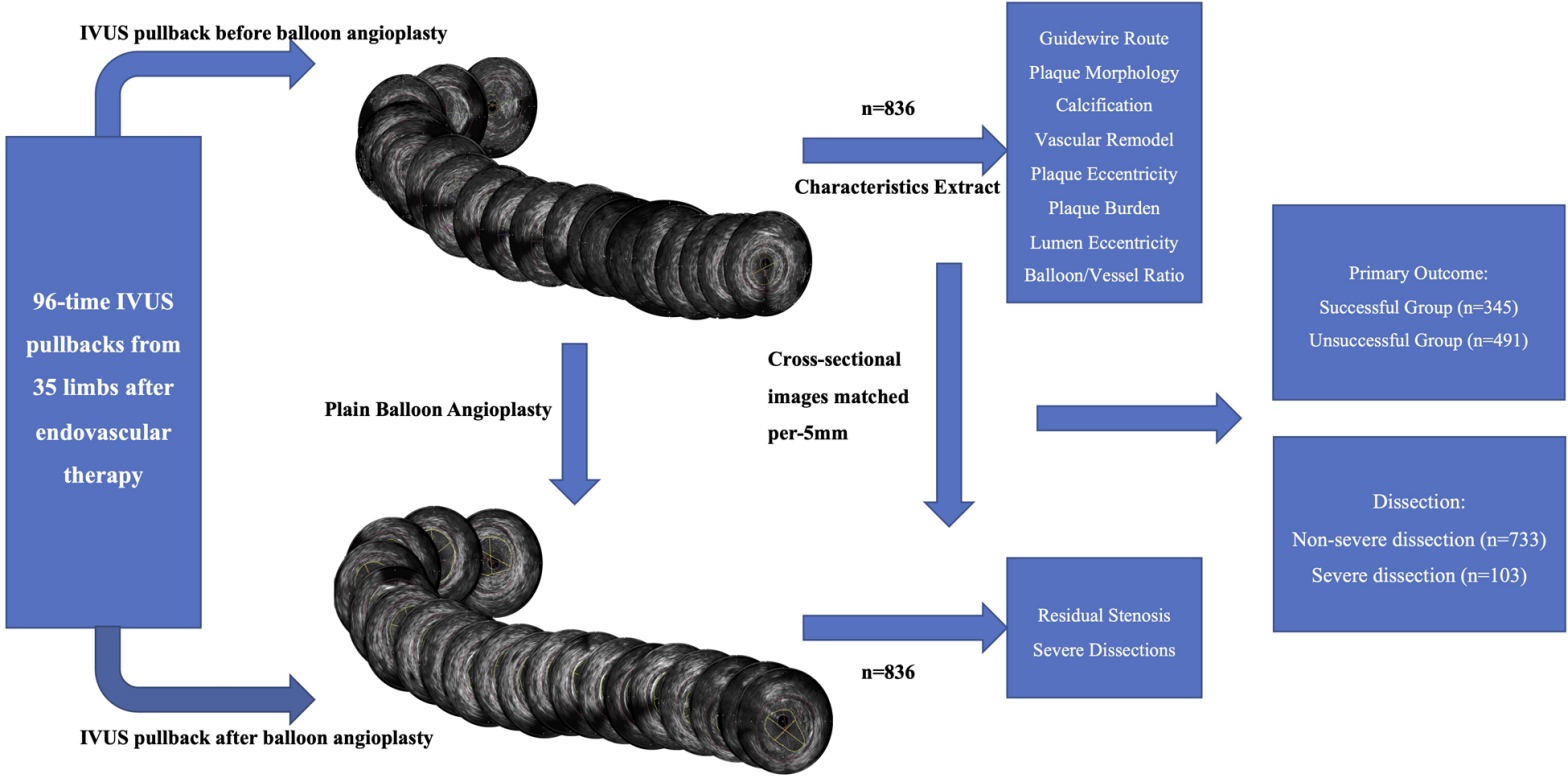
Flow chart of IVUS division and cross-sectional image analysis. Schematic representation of the classification of 836 cross-sectional images into successful (*n* = 345), unsuccessful (*n* = 491), severe dissection (*n* = 103), and non-severe dissection (*n* = 733) groups per reference standards to analyze the risk factors for severe dissections. IVUS, intravascular ultrasound.

This retrospective, observational study included 35 limbs from 34 consecutive patients enrolled between September 2020 and February 2022. Interventional procedures were performed, and 96 runs (including pre- and post-angioplasty) of the IVUS catheter (OptiCross 40 MHz, Boston Scientific, USA) were performed. IVUS pullbacks were divided per 5 mm length into 952 cross-sectional images in the femoropopliteal artery with the IVUS imaging system; 836 cross-sectional images met the criteria and were divided into successful (*n* = 345) and unsuccessful (*n* = 491) groups per reference standard. Cross-sectional images were also divided into severe dissection (*n* = 103) and non-severe dissection (*n* = 733) groups to analyze the risk factors for severe dissections (Figure 1).

This study complied with the principles of the Declaration of Helsinki for investigation in humans. Our ethics committee approved it (approval no. 2021-088), and each patient provided written informed consent before the procedure.

### 2.3. Interventional procedures

The following precautions were taken to ensure that the ultrasound images obtained before and after balloon angioplasty corresponded to each other: (1) a 100 cm radiopaque ruler was used to locate target segments and IVUS catheter markers, allowing immediate comparison of the IVUS and angiography images; (2) to eliminate parallax, IVUS catheter markers were centered on the screen during fluoroscopy; and (3) the calcifications observed in the pre- and post-angioplasty intravascular ultrasound images were matched.

After local anesthesia with 2.0% lidocaine, a 6.0- or 7.0 F guiding sheath was inserted into the affected area. Based on the disease characteristics, the surgeon decided on the most appropriate technique, contralateral approach or anterograde puncture. A 0.018- or 0.014 inch guidewire was used for the intraplaque approach. In cases where the intraplaque crossing was unsuccessful, a subintimal crossing using a 0.018- or 0.035 inch guidewire was attempted. Angiography and IVUS pullbacks were performed before and after balloon angioplasty. Each pullback of the IVUS probe was controlled using an automatic pullback device at a speed of 1 mm/s. The IVUS catheter was passed across the diseased site and stopped on the healthy segment (at least 5 mm from the distal end of the diseased area); in cases where the IVUS catheter failed to cross the diseased area, pre-inflation using a 2 mm balloon was used to assist catheter advancement. Using angiography, the largest balloon size was adapted to the reference vessel diameter, with a balloon-to-vessel ratio of 1:1. Balloon inflation was performed for ≥120 s. Adjunctive stent implantation was required after balloon angioplasty in cases where flow-limiting dissection or residual stenosis >30% was observed on angiography images. The drug-coated balloon was used at the operator's discretion. After the procedure, low-molecular-weight heparin was used for 3 days, and patients were administered clopidogrel 75 mg or aspirin 100 mg daily for 1 year.

### 2.4. IVUS images definition and analysis

All IVUS cross-sectional images were analyzed using the Polar Viewer system (Boston Scientific, USA) and Radiant Dicom Viewer (Version 5.4, Medixant, Poland). Primary successful balloon angioplasty was defined as the absence of severe dissection or residual stenosis <30% (1). Measurements were taken relative to the center of the lumen rather than the IVUS catheter center. Vascular surgeons and cardiologists blinded to the clinical and procedural data analyzed all images. The operator in the analysis reviewed disagreements regarding the interpretations of IVUS or angiography images.

**Figure 2:**
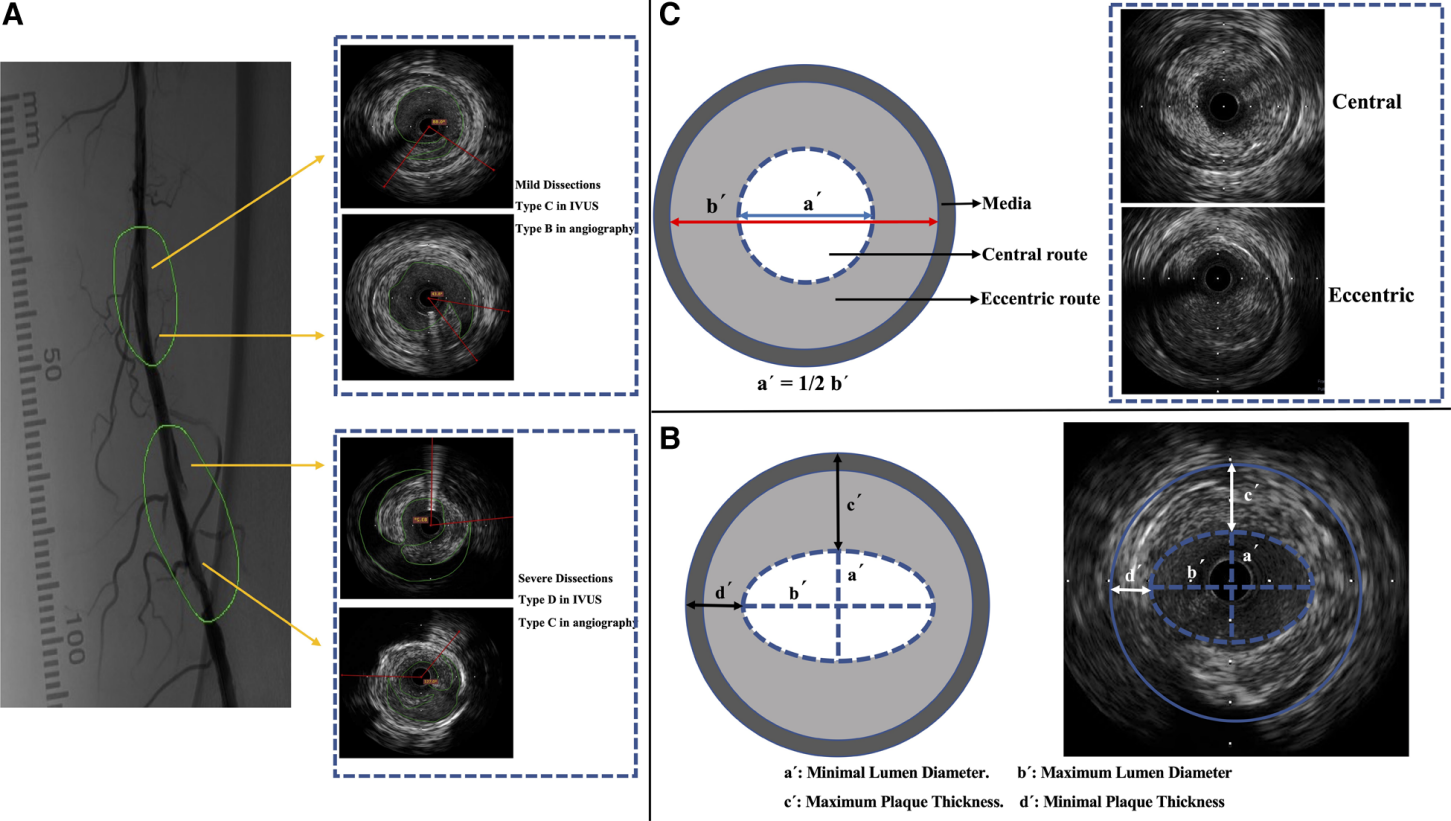
IVUS imaging and characterization. (**A**) Classification of dissections in IVUS images (according to the classification by Honye et al.) (14) and in angiography (according to the National Heart, Lung, and Blood Institution classification) (15). (**B**) Measurement of lumen diameter and plaque thickness. (**C**) Definition of guidewire route. IVUS, intravascular ultrasound.

Dissection severity was determined using the IVUS categorization developed by Honye et al. after coronary balloon angioplasty (14). When dissection based on IVUS image was defined as type D-Type E2, proven to correlate with severe angiographic dissection (12), the segment was reviewed in angiography according to the National Heart, Lung and Blood Institution classification (15). If the cross-sectional and angiography images (type C or above classification) simultaneously showed severe dissection, the IVUS cross-sectional image was confirmed to be showing a severe dissection (Figure 2A).

Residual stenosis on IVUS cross-sectional images was estimated by conflating the diameters, residual stenosis = (reference lumen diameter − average lumen diameter after balloon angioplasty)/reference vessel diameter.

EEM appeared as a highly echoic layer between the media and adventitia. In the cross-sectional image analysis, EEM, which could be measured more accurately than the internal elastic membrane (IEM), was used to evaluate vessel size and diameter (10). However, the IEM could not be clearly separated.

The proximal reference was the largest lumen site closest to the stenosis in the same segment (within 10 mm of the stenosis); the distal reference was the largest lumen site farthest from the stenosis in the same segment (within 10 mm of the stenosis). The average lumen sizes at the proximal and distal reference sites were used to calculate the average reference lumen size. The minimum lumen diameter was the smallest at the center of the lumen; the maximum lumen diameter was the widest at the center of the lumen (Figure 2B). The average lumen diameter was calculated as the average of the minimum and maximum lumen diameters. The reference diameter was the average lumen diameter of the reference cross-sectional images (16).

The increase or decrease in the EEM area during the development of atherosclerosis is referred to as vascular remodeling. An index that describes the remodeling magnitude and direction was expressed as the EEM area of the cross-sectional image/reference vessel size.

An index value of >1.0 represents positive remodeling. This study defined positive or negative remodeling as a >10% increase or decrease in the index value, respectively (17). Lumen eccentricity = (maximum lumen diameter − minimum lumen diameter)/maximum lumen diameter (Figure 2B) (16). Plaque burden measurements were independent of luminal area stenosis. Therefore, the plaque burden represents the area within the EEM occupied by the plaque, regardless of the lumen compromise. Plaque burden = (EEM area − lumen area)/EEM area.

Maximum plaque thickness: the longest distance from the intimal leading edge to the EEM along any line passing through the lumen center. Minimum plaque thickness: the shortest distance forms the intimal leading edge of the EEM along any line passing through the lumen center. Plaque eccentricity = (maximum plaque thickness − minimum plaque thickness)/maximum plaque thickness. Eccentric plaques were sized >0.5, and centripetal plaques were sized ≤0.5 (Figure 2B) (16).

According to a study by Takenobu et al., the guidewire route was characterized as either guidewire transit via the inner half of the lumen radius (central wiring) or guidewire passage through the outer half of the luminal radius (eccentric wiring) (Figure 2C) (12). Soft plaques, fibrous plaques, mixed plaques, and calcified plaques were classified (10), and calcification severity was classified as none, mild (25% arc), moderate (25%–50%), or severe (>50%) (1). The balloon/vessel size ratio was determined. The ratio is the cross-sectional area of the balloon catheter dilated at the nominal pressure that divided the EEM region.

### 2.5. Statistical analysis

Continuous data were tested using the *t*-test or Mann–Whitney *U*-test and are reported as mean ± standard deviation or median (interquartile range), depending on distribution normality. Categorical variables are presented as numbers (percentages) and were compared using the chi-square test or Fisher's exact test. The risk factors for failed balloon angioplasty and severe dissection on IVUS were determined using multivariate logistic regression analysis. Independent variables are expressed as odds ratios (ORs) with 95% confidence intervals (CIs). Additionally, the variance inflation factor was tested to ensure non-significant collinearity between factors. Statistical significance was set at *p* < .05. SPSS software (version 24; IBM Crop) and R software (version 4.2.0, https://www.R-project.org/), R package “CBCgrps” (17), and R package “rms” were used to conduct statistical analyses, and R package “forest” was used for visual multivariable regression results.

## 3. Results

### 3.1. Baseline clinical and procedural characters

Chronic total occlusion was observed in 20 of the 35 lesions (57.1%), and 14 target lesions in the popliteal artery (40.0%) were included. Nine limbs showed critical limb ischemia; most patients were administered antiplatelet agents before the procedure. The mean lesion length was 115.8 mm, measured by angiography; 17.1% of patients were treated with a drug-coated balloon and 62.9% required remedial stents, owing to flow-limiting dissections or high residual stenosis after balloon angioplasty (Table 1).

**Table 1:** Baseline clinical, procedural, and angiographic characteristics of 35 femoropopliteal lesions.

Variables	Limbs (*n* = 35)
Baseline clinical characteristics	
Age (years)	74.1 ± 8.1
Male	25 (68.6)
Lesion location	
SFA	21 (60.0)
SFA and popliteal artery (P1)	14 (40.0)
Lesion type	
Stenosis	15 (42.9)
Chronic total occlusion	20 (57.1)
Pre-ABI	0.36 ± 0.08
Post-ABI	0.75 ± 0.08
Critical limb ischemia	9 (25.7)
BMI (kg/m^2^)	23.15 ± 3.3
Rutherford classification	
2	0 (0.0)
3	16 (45.7)
4	18 (51.4)
5	1 (2.9)
6	0 (0.0)
Right limb	22 (62.9)
Smoker	21 (60.0)
Alcohol history	11 (31.4)
Hypertension	24 (68.6)
Diabetes mellitus	16 (45.7)
Hyperlipidemia	15 (42.9)
Coronary artery disease	10 (28.6)
Renal dysfunction	2 (5.7)
Cerebrovascular disease	2 (5.7)
Aspirin (100 mg/day)	19 (54.3)
Cilostazol (75 mg/day)	12 (34.3)
Anticoagulant	10 (28.6)
Angiographic and procedural characters	
TASC classification	
A	0 (0.0)
B	11 (31.4)
C	19 (54.3)
D	5 (14.3)
Run-off vessel	
0	3 (8.6)
1	17 (48.6)
2	15 (42.9)
3	0 (0.0)
Lesion length (mm)	115.8 ± 26.8
Reference diameter (mm)	5.1 ± 0.64
Balloon size (mm)	5.2 ± 0.66
Balloon inflation time (s)	149.7 ± 26.0
DCB	6 (17.1)
Bare-mental stent	22 (62.9)

Data are reported as numbers (percentages) or mean ± standard deviation. SFA, superficial femoral artery; ABI, ankle branchial pressure index; BMI, body mass index; DCB, drug-coated balloon.

### 3.2. IVUS cross-sectional imaging outcomes

In 836 matched cross-sectional images, including 1,672 vessel slices, 61.1% crossed the segment with the central route; additionally, the mean lumen eccentricity was 0.26, reflecting the luminal shape. Most images showed fibrous plaques (41.6%) or soft plaques (29.2%) in terms of morphology. No calcification was detected in the upper half of the images (55.5%), and negative vascular remodeling was most frequently observed (43.7%). The mean plaque burden before balloon angioplasty was 0.73, and most images showed eccentric plaques (73.1%) (Table 2).

**Table 2:** Baseline morphology characteristics of IVUS images.

Variables	Cross-sectional images (*n* = 836)
Guidewire route	
Central	511 (61.1)
Eccentric	325 (38.9)
Plaque morphology	
Calcified	63 (7.5)
Fibrous	348 (41.6)
Mixed	181 (21.7)
Soft	244 (29.2)
Severity of calcification	
None	464 (55.5)
Mild (<25%)	246 (29.4)
Moderate (25%–50%)	84 (10.0)
Severe (>50%)	42 (5.0)
Vascular remodel	
Negative (<0.9)	365 (43.7)
None (0.9–1.1)	217 (26.0)
Positive (>1.1)	254 (30.4)
Plaque eccentricity	
Centripetal (≤0.5)	225 (26.9)
Eccentric (>0.5)	611 (73.1)
Plaque burden	0.73 ± 0.15
Lumen eccentricity	0.26 ± 0.15
Balloon/vessel ratio	0.73 ± 0.19

Data are reported as numbers (percentages) or mean ± standard deviation. IVUS, intravascular ultrasound.

### 3.3. Univariable analysis outcomes

A total of 491 cross-sectional images showed unsuccessful balloon angioplasty; 345 images showing successful primary balloon angioplasty were included in the univariable analysis. Higher plaque burden (plaque characteristics) was noted in the unsuccessful group [0.72 (0.57, 0.81) vs. 0.78 (0.68, 0.86), *p* < .001]. A significant difference was noted in vascular remodeling (luminal characteristics) between the groups (*p *< .001) with significantly higher lumen eccentricity in the unsuccessful group [0.21 (0.12, 0.32) vs. 0.24 (0.16, 0.35), *p *< .001]. Conversely, the unsuccessful group showed a lower balloon/vessel ratio than the successful group [0.74 (0.64, 0.83) vs. 0.71 (0.57, 0.84), *p *< .01] (Table 3).

**Table 3:** Univariable analysis for predictive factors for primary success and severe dissection.

Variables	Total (*n* = 836)	Primary success			Dissection		
		Success (*n* = 345)	Unsuccess (*n* = 491)	*p*	Non-severe dissection (*n* = 733)	Severe dissection (*n* = 103)	*p*
Guidewire route				.16			**<**.**001**
Central	511 (61.12)	221 (64.06)	290 (59.06)		465 (63.44)	46 (44.66)	
Eccentric	325 (38.88)	124 (35.94)	201 (40.94)		268 (36.56)	57 (55.34)	
Plaque morphology				.58			.57
Calcified	63 (7.54)	27 (7.83)	36 (7.33)		58 (7.91)	5 (4.85)	
Fibrous	348 (41.63)	134 (38.84)	214 (43.58)		304 (41.47)	44 (42.72)	
Mix	181 (21.65)	77 (22.32)	104 (21.18)		161 (21.96)	20 (19.42)	
Soft	244 (29.19)	107 (31.01)	137 (27.9)		210 (28.65)	34 (33.01)	
Calcification				.77			.75
None	464 (55.5)	195 (56.52)	269 (54.79)		406 (55.39)	58 (56.31)	
Mild	246 (29.43)	95 (27.54)	151 (30.75)		213 (29.06)	33 (32.04)	
Moderate	84 (10.05)	37 (10.72)	47 (9.57)		76 (10.37)	8 (7.77)	
Severe	42 (5.02)	18 (5.22)	24 (4.89)		38 (5.18)	4 (3.88)	
Vascular remodel				**<**.**001**			.23
Negative	365 (43.66)	129 (37.39)	236 (48.07)		314 (42.84)	51 (49.51)	
None	217 (25.96)	87 (25.22)	130 (26.48)		189 (25.78)	28 (27.18)	
Positive	254 (30.38)	129 (37.39)	125 (25.46)		230 (31.38)	24 (23.3)	
Plaque eccentricity				.68			.14
Centripetal	225 (26.91)	96 (27.83)	129 (26.27)		204 (27.83)	21 (20.39)	
Eccentric	611 (73.09)	249 (72.17)	362 (73.73)		529 (72.17)	82 (79.61)	
Plaque burden	0.76 (0.64,0.84)	0.72 (0.57,0.81)	0.78 (0.68,0.86)	**<**.**001**	0.75 (0.64,0.84)	0.77 (0.68,0.84)	.40
Lumen eccentricity	0.23 (0.14,0.33)	0.21 (0.12,0.32)	0.24 (0.16,0.35)	**<**.**001**	0.23 (0.14,0.33)	0.24 (0.16,0.36)	.31
Balloon/vessel ratio	0.72 (0.6, 0.83)	0.74 (0.64,0.83)	0.71 (0.57,0.84)	**<**.**01**	0.72 (0.59,0.83)	0.75 (0.64,0.89)	.**04**

Data are reported as numbers (percentages), mean ± standard deviation, or median (interquartile range). Bold represents statistically significant difference, or *p* < .05.

A total of 103 cross-sectional images (12.3%) showed severe dissection, and 733 images (87.7%) did not. In the univariable analysis, a more eccentric cross was noted in the severe dissection group (55.3% vs. 36.6%, *p *< .001), showing a higher balloon/vessel ratio than that in the non-severe dissection group [0.75 (0.64, 0.89) vs. 0.72 (0.59, 0.83), *p *= .04] (Table 3).

### 3.4. Predictors of failed balloon angioplasty and severe dissection

**Figure 3:**
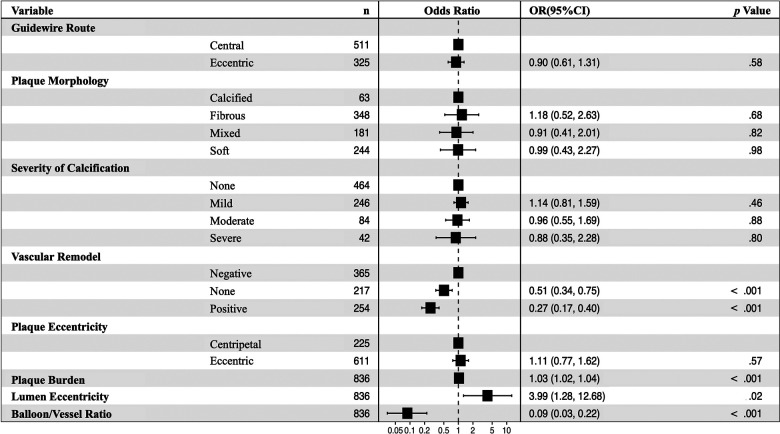
Multivariable analyses for predictors of failed balloon angioplasty in 836 images. Non-vascular remodeling (OR: 0.51, 95% CI: 0.34–0.75, *p* < .001), positive vascular remodeling (OR: 0.27, 95% CI: 0.17–0.40, *p* < 001), and balloon/vessel ratio (OR: 0.09, 95% CI: 0.03–0.22, *p* < .001) were protective factors for unsuccessful balloon angioplasty. CI, confidence interval, OR, odds ratio.

In the multivariate analysis, plaque burden (OR: 1.03, 95% CI: 1.02–1.04, *p* < .001) and lumen eccentricity (OR: 3.99, 95% CI: 1.28–12.68, *p* = .02) were shown to be risk factors for unsuccessful balloon angioplasty. Further, non-vascular remodeling (OR: 0.51, 95% CI: 0.34–0.75, *p* < .001), positive vascular remodeling (OR: 0.27, 95% CI: 0.17–0.40, *p* < 001), and balloon/vessel ratio (OR: 0.09, 95% CI: 0.03–0.22, *p* < .001) were protective factors for unsuccessful balloon angioplasty (Figure 3).

**Figure 4:**
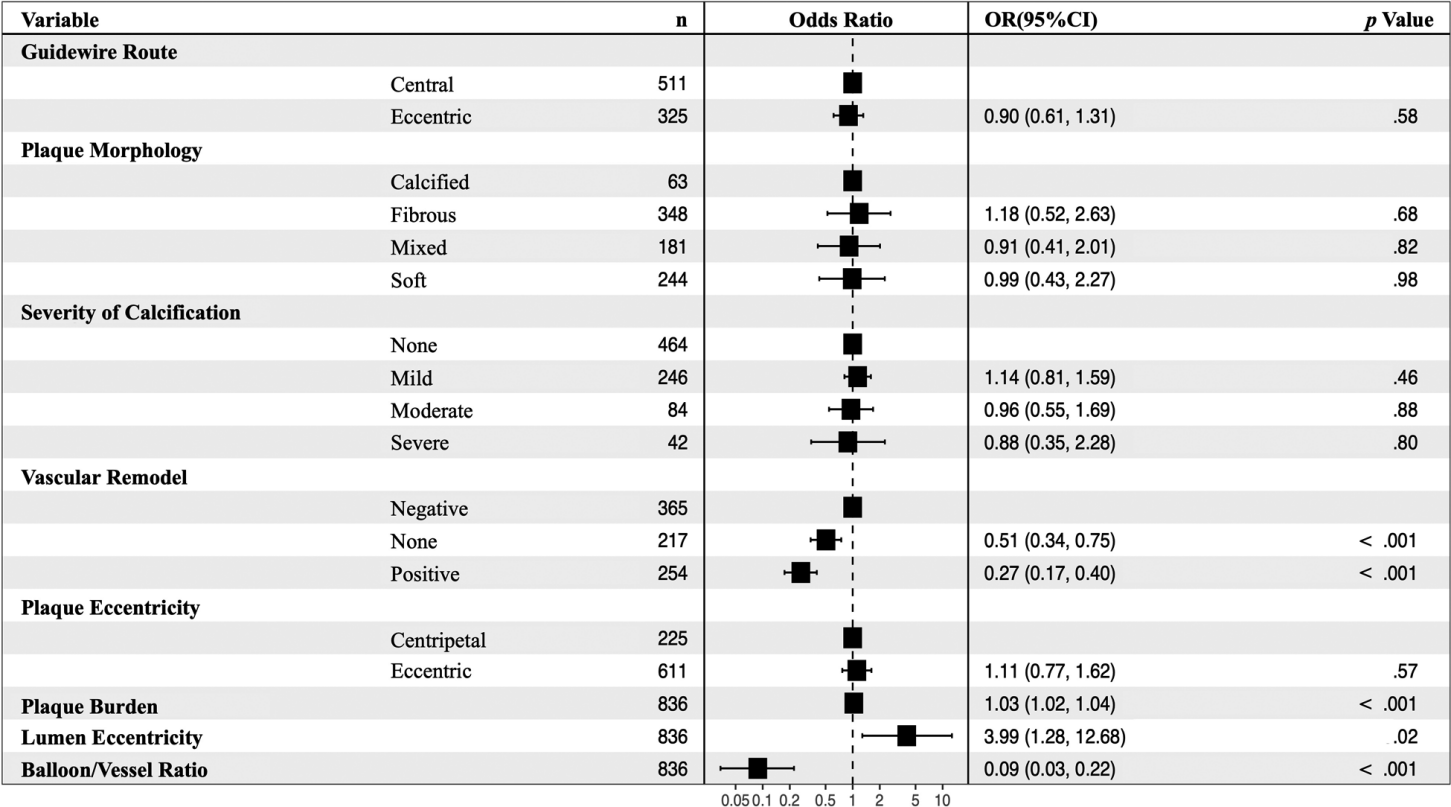
Multivariable analyses for predictors of severe dissection after balloon angioplasty in 836 images. In the non-severe and severe dissection groups, the eccentric guidewire route (OR: 2.10, 95% CI: 1.22–3.65, *p* = .01) was an independent risk factor for severe dissections after balloon angioplasty. CI, confidence interval, OR, odds ratio.

The eccentric guidewire route (OR: 2.10, 95% CI: 1.22–3.65, *p* = .01) was an independent risk factor of severe dissections after balloon angioplasty (Figure 4). Each factor was tested using variance inflation. Factor and collinearity diagnostic tests did not reveal significant collinearity factors in multivariate regressions.

## 4. Discussion

This study aimed to clarify the risk factors for failed balloon angioplasty and severe dissection. The eccentric guidewire route was noted to be an independent risk factor for severe dissections, similar to previously published findings from Japan indicating that the central guidewire route was a protective factor for severe dissections (12). Here, an “eccentric guidewire route” indicated that the guidewire closed the IEM. Although some images showed the subintimal and intramedial cross, most were excluded because they were incomplete images with an angle of <270° (23 of 116 excluded images).

During endovascular treatment, a guidewire leads the balloon catheter to the target lesion, and uniform pressure is applied to the plaque and vessel around the balloon catheter if the catheter is in the central route. In contrast, eccentric inflation of the balloon catheter results in non-uniform compression, and in some cases of our study, dissection even involves injury to the media or adventitia (18). The nearer the balloon is to the IEM, the greater the rigidity of the vessel wall and the tendency of unequal region stress between the plaque and IEM regions; this results in an increased risk of disruption from longitudinal shearing with balloon dilatation (6, 19, 20). Some studies have indicated that vessel calcification is associated with an additional increased risk of postprocedural flow-limiting dissections based on angiography findings (5); however, a study by Hanbee et al. indicated that severe calcification was a protective factor against severe dissection (21). Our study did not show any significant statistical difference in this regard; this may be due to the limited number of participants with severe calcification (5.0%); additionally, some cross-sectional images with thick calcification were excluded from influent measurements (17 of 116 excluded images). Further studies that include more calcified segments should be conducted to clarify the influence of severe calcifications.

Second, we analyzed the risk factors for unsuccessful balloon angioplasty, indicated by severe dissection or high residual stenosis. Residual stenosis plays an essential role in primary balloon angioplasty failure in some cases; however, many studies attributed greater priority to severe dissection-associated balloon angioplasty failure. Our study calculated residual stenosis using the diameter to match the angiography standard, and in all cross-sectional images with severe dissections, the diameter was measured in the lumen without including the blood flow in the dissection segment; therefore, most images of severe dissections showed high residual stenosis (93 of 103 severe dissections) (22).

Balloon angioplasty is hypothesized to work by causing permanent distortion of the artery walls through intima cracking or dehiscence and stretching of the arterial wall, as observed in animal models and cadaveric vessels (23–25). Therefore, significantly enhanced vascular patency, requiring adequate atheromatous material redistribution, compression, and elastic vessel wall expansion, is necessary. The present study indicated that plaque burden, which refers to the vessel plaque area, was a risk factor for unsuccessful balloon angioplasty. Some studies have reported that most of the luminal gain is caused by adventitial and medial stretching but not from plaque compression (26). Therefore, a lesion with a higher plaque burden would show higher residual stenosis after balloon angioplasty. Moreover, lumen eccentricity indicating the luminal shape was noted as a risk factor detected in this study. Higher lumen eccentricity corresponded to increased luminal shape nonuniformity, usually presented as a thinner lumen (Figure 2B). It was observed that high residual stenosis was related to the elastic recoil of the arterial wall in balloon angioplasty, and the only variable associated with the elastic recoil magnitude is a temporary stretch, as previously reported (27). High lumen eccentricity causes non-uniform pressure on the arterial wall, leading to higher elastic recoil after balloon inflation because of a preserved segment of the media (28). The poor correlation between angiographic and ultrasound lumen dimensions after angioplasty may explain the lack of correlation between plaque composition or morphology and the magnitude of the elastic recoil that causes residual stenosis (29).

Some protective factors were also identified in this study. Non-negative vascular remodeling decreases the risk of unsuccessful balloon angioplasty. During balloon angioplasty, compensatory enlargement of the artery in response to plaque formation helps preserve the luminal area. In contrast, arterial shrinkage accelerates lumen narrowing through plaque formation. Some studies have indicated that plaque size and the mode and degree of arterial remodeling indicate the severity of lumen narrowing (19, 30). In the lesion segment in the limb, non-negative remodeling may lead to less elastic recoil than that noted in the negative remodeling cross-section. The balloon diameter was selected according to the disease-free segment; when the balloon was inflated, overextensions was performed in the shrunken arterial wall in contrast to that in the non- or positive remodeling cross-section. Our study showed results similar to those reported by Gerard et al., indicating that less stretch of the arterial wall and improved plaque burden decrease were observed in arterial cross sections that had undergone compensatory enlargement than that in those that had undergone arterial wall shrinkage (22). The balloon/vessel ratio was noted to be a protective factor, confirming that the larger the size of the balloon, the lower the residual stenosis. This may be due to a smaller vessel size than the actual vessel size as assessed using angiography. Consequently, the balloon size chosen for angiography would be smaller in size than the one chosen based on IVUS images, leading to insufficient plaque compression and arterial wall stretching. Therefore, larger balloons may result in less residual stenosis. The study by Van Erven et al. using rabbit models showed that oversizing the balloon relative to the local artery size resulted in increased medial necrosis, subsequently decreasing the elastic recoil of media (31). This explains the mechanism underlying high balloon/vessel ratios leading to increased luminal gain. However, only 4–6 mm balloons were used in the present study, and it was difficult to determine the threshold for maximal elastic recoil. Further studies should investigate the best fit of the balloon/vessel ratio that could enlarge the lumen to its highest limit without severe elastic recoil and dissection.

Although IVUS is not as commonly used in peripheral arterial disease as it is in coronary arterial disease, it is necessary to efficiently assess the morphologies of plaque, lumen, and arterial walls in peripheral arterial disease. In many cases, intravascular conditions are more complex and unpredictable in peripheral arteries than in coronary arteries. During interventional procedures of the femoropopliteal artery, according to our results, balloon angioplasty tends to fail with high risk, and thus, other treatment strategies including debulking should be performed to avoid high residual stenosis and severe dissections. Further studies should explore invasive and non-invasive imaging and hemodynamic characteristics collectively to clarify intravascular conditions before and after balloon angioplasty and to precisely choose treatment strategies to improve patients' long-term outcomes.

### 4.1. Limitations

This study has some limitations that should not be ignored. First, the IVUS data were retrospectively collected from a single center. Second, our study analyzed cross-sectional images of lesions but could not analyze longitudinal sections to characterize factors such as the length of the target lesion or calcification, which influences the outcome of balloon angioplasty (32). Images of thick calcification and subintimal and intramedial crossing were excluded due to incomplete imaging and a shaded elastic membrane. Third, there was no standard classification of dissection using IVUS (33). Although we combined IVUS images and angiography to confirm severe dissection, there was a risk of missed dissections. Additionally, although the relationship between dissection or residual stenosis in coronary disease and characteristics extracted from IVUS were reported, they were uncommon in peripheral arterial disease. Thus, these characteristics require further investigation.

## 5. Conclusion

Plaque burden and luminal eccentricity were risk factors for unsuccessful balloon angioplasty, and the eccentric guidewire route was an independent risk factor for severe dissection after balloon angioplasty.

## Data Availability

The raw data supporting the conclusions of this article will be made available by the authors, without undue reservation.
